# Comparing the effects of Pilates, corrective exercises, and Alexander’s technique on upper cross syndrome among adolescent girls student (ages 13–16): a six-week study

**DOI:** 10.1186/s13102-024-00933-2

**Published:** 2024-06-28

**Authors:** Nesa Shadi, Karim Khalaghi, Mohammad Seyedahmadi

**Affiliations:** 1 Department of Sport Sciences, Hakim Nizami Qochan Institute of Higher Education, Quchan, Khorasan Razavi Province Iran; 2 Department of Sport Sciences, Velayat University, Iranshahr, Iran

**Keywords:** Kyphosis, Forward head, Musculoskeletal abnormalities, Physical activity, Exercise therapy, Adolescent

## Abstract

**Introduction:**

Upper Cross Syndrome is a pattern of muscle imbalance and postural dysfunction that can cause discomfort and pain. This study’s objective was to compare the effects of Pilates exercises, corrective exercises, and Alexander’s technique on upper cross syndrome in adolescent girls aged 13–16 years: a six-week intervention study.

**Methods:**

The present study was Quasi-experimental, and its statistical population consisted of 13 to 16-year-old female students. Forty-five students who were diagnosed with upper cross syndrome were purposefully selected as samples and randomly assigned to three groups: Pilates exercises (*N* = 15), corrective exercises (*N* = 15), and Alexander’s technique (*N* = 15). The participants performed exercises for 60 min per session, three sessions per week, and six weeks. This study’s objective was to compare the effects of Pilates exercises, corrective exercises, and Alexander’s technique on upper cross syndrome in adolescent girls aged 13–16 years: a six-week intervention study. This study was retrospectively registered in the Iranian Registry of Clinical Trials (IRCT) on 2023-09-19 to comply with the journal’s policies. The assigned trial registration number is IRCT20230810059106N1.

**Results:**

The results of the dependent t-test showed significant decreases in forward head angle (*p* = 0.0001), rounded shoulder (*p* = 0.001), and kyphosis (*p* = 0.0001) as a result of corrective exercises. There were also significant decreases in forward head angle (*p* = 0.0001), rounded shoulder (*p* = 0.002), and kyphosis (*p* = 0.001) after six weeks of practising Alexander’s technique. However, in the case of Pilates exercises, a significant decrease in forward head angle (*p* = 0.110), rounded shoulder (*p* = 0.598), and kyphosis (*p* = 0.371) was not observed. The one-way analysis of variance revealed a significant difference in the forward head angle (*p* = 0.012), rounded shoulders (*p* = 0.013), and kyphosis (*p* = 0.009).

**Conclusions:**

The effect of Alexander’s technique and corrective exercises on forward head angle, rounded shoulder, and kyphosis abnormalities was almost similar and more effective than pilates exercises.

**Supplementary Information:**

The online version contains supplementary material available at 10.1186/s13102-024-00933-2.

## Introduction

The widespread use of electronic devices like mobile phones has become essential in daily life, especially for students [1]. Prolonged use of these devices can lead to abnormal postures, causing neuromuscular imbalances and musculoskeletal conditions [2] such as upper crossed syndrome (UCS). UCS is characterized by weakened and tight muscles [3], often leading to symptoms like forward head posture and rounded shoulders [4]. These abnormal postures can also affect lung capacity and respiratory efficiency, leading to decreased exercise tolerance [5].

Pilates exercises(PE) are a modern training method that concentrates on the body, particularly the core muscles, pelvis, spine, and UCS [6]. Various studies have examined the effect of PE on lumbar lordosis and dorsal kyphosis [7, 8]. Pilates facilitates flexibility, strength, and overall body control and endurance improvements. This exercise form also focuses on body alignment, developing a strong core area, and enhancing coordination and balance. UCS is essential for maintaining body stability [9]. One of the primary methods for correcting abnormal spinal curvatures is through corrective exercises (CE), which can effectively improve alignment [10, 11]. The Corrective Exercise Specialization (CES) program by the National Academy of Sports Medicine (NASM) equips fitness professionals to evaluate and address movement dysfunctions, muscular imbalances, and posture-related concerns in their clients [11]. Studies have shown that CE based on NASM principles can improve abnormal spinal curvatures [12], and teaching proper posture can significantly reduce these abnormalities [13, 14]. The Alexander Technique (AT) is a widely practiced physical method that aims to increase body awareness and improve movement patterns [15]. The goal of the AT is to assist individuals in increasing body awareness and using this information to achieve more efficient functioning. It utilizes a teacher-student model to enable the student to become aware of their body’s sensory-motor condition and to change their habitual faulty movement patterns. The primary focus of this technique is on education rather than treatment [16]. It has been shown to be highly effective in improving balance and is recommended for individuals with UCS to enhance their overall balance and well-being [17].

Therapists have employed several methods to treat UCS, including muscle energy techniques [18], Kinesio taping [19], stretching exercises [20], pressure biofeedback [21], and other procedures. Although these methods have demonstrated effectiveness in clinical settings, they have some drawbacks. A trained rehabilitation therapist must administer the treatment; patients cannot perform it independently. Additionally, the costs associated with these treatments can be high and may not be feasible for some patients. In general, designing an effective method of treatment for this issue contributes significantly to saving a considerable portion of healthcare costs. The researchers focused on 13- to 16-year-old girls for several reasons. First, UCS is more common in girls than boys, and its prevalence increases during adolescence. Second, the teenage years in girls are a time of rapid physical and hormonal changes. These changes can affect the alignment of the spine and cause musculoskeletal problems. Third, UCS can impact posture, balance, and overall health. It can also lead to pain, headaches, and other health problems in teenage girls. Additionally, considering the prevalence of UCS among teenage girls due to improper use of mobile phones, tablets, and computers, not adhering to proper principles during walking, sitting on chairs, lying down, and carrying heavy backpacks highlights the importance of conducting this research. The study is critical because it will provide valuable information on the effectiveness of PE, CE, and AT in improving UCS among adolescent girls. UCS is a common condition that can cause pain, discomfort, and poor posture, characterized by RS, FH, and increased thoracic spine curvature. PE, CE, and AT can improve posture and reduce pain. The study aims to compare the effectiveness of these three methods in improving UCS among adolescent girls and determine which is most effective for improving posture and reducing pain in this population. This study is significant as no previous study has compared the effects of PE, CE, and AT on UCS in teenage girls. Therefore, this study aims to investigate whether six weeks of PE, CE, and AT can affect UCS differently in 13- to 16-year-old girls.

## Methods

### Study design and participants

The trial protocol for this study was retrospectively registered in the Iranian Registry of Clinical Trials on 2023-09-19 to comply with the journal’s policies, with the assigned approval number IRCT20230810059106N1. This research was Quasi-experimental, and its statistical population consisted of 13 to 16-year-old female students. While this design does not strictly adhere to CONSORT guidelines for randomized controlled trials, we have endeavored to report our methods and findings transparently and comprehensively.

### Group allocation

**Fig. 1:**
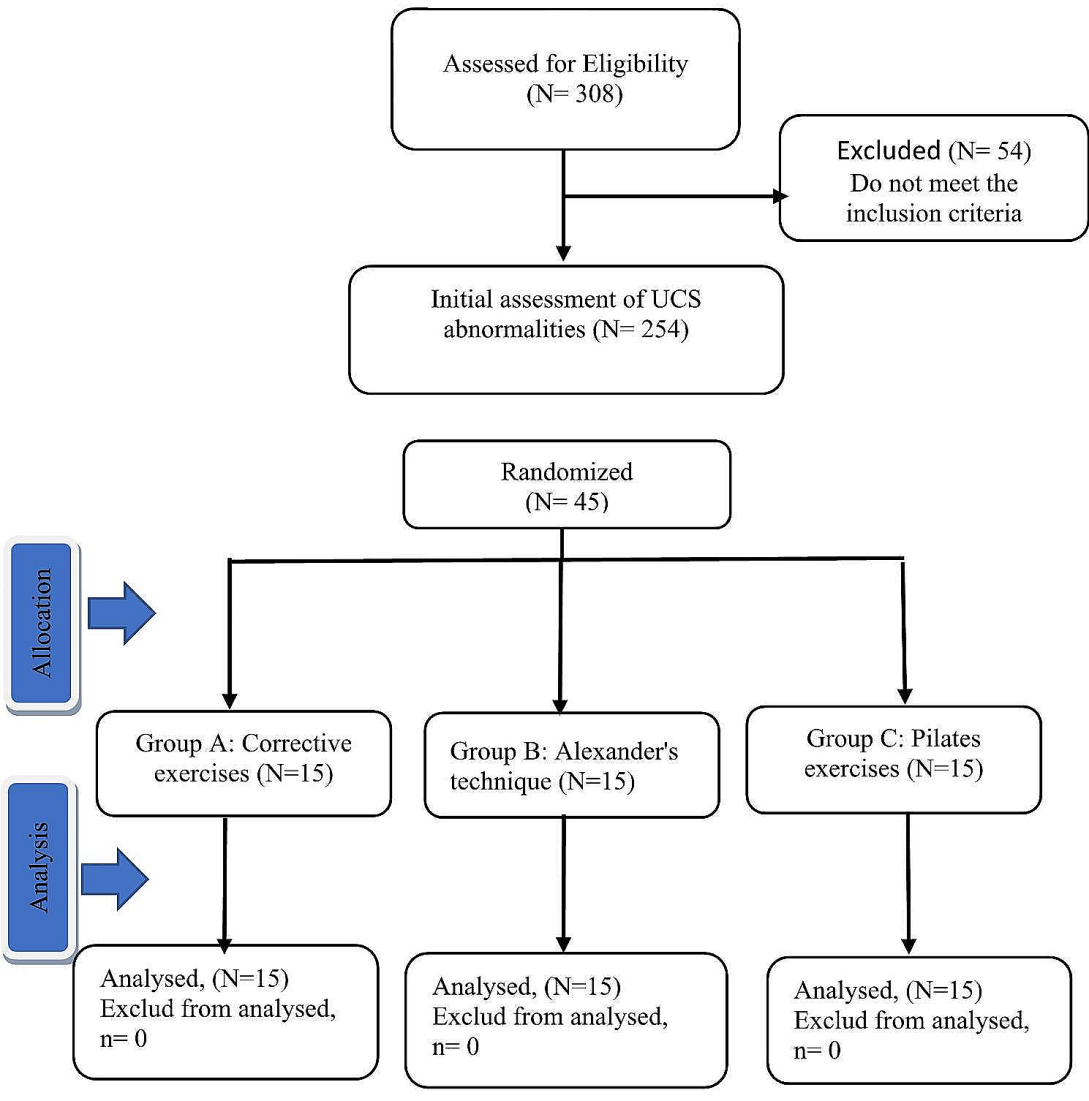
Consort fow chart for enrollment and intervention

Among the ten first—and second-grade girls’ high schools in the city of Quchan(3000 students), a cluster random sampling approach was employed to select three schools (1000 students). Of the total, 308 students volunteered to participate in the research, with their parents completing the informed consent forms. Subsequently, students between 13 and 16 in these schools underwent individual assessments for UCS. Forty five students who were diagnosed with UCS selected as samples and randomly assigned to three groups: PE (*N* = 15), CE (*N* = 15), and AT (*N* = 15). The participants performed CE, AT, and PE for 60 min per session, three sessions per week, and for 6 weeks that presented in Consort fow chart (Fig. 1). PE and CE exercises were done in the gym and AT exercises were done in the home.

### Inclusion and exclusion criteria

Participants in the study had to to simultaneously have postural abnormalities such as kyphosis, FH, and RS, and express a willingness to participate. Those with signs of illness, fractures, surgeries, joint problems, injuries in the spine, skeletal-muscular imbalances, lower limb cross syndrome, abnormal BMI, or engaging in regular physical activity for at least 6 h per week were excluded.

### Measurement of the kyphosis, rounded shoulder and forward head

The Corrective Exercise Specialist conducted the measurements.

Thoracic kyphosis angle was measured with a flexicurve ruler, 50 cm in length and 2 cm wide, with the spinous processes of the T_2_ and T_12_ vertebrae serving as the starting and ending points [22, 23]. To locate the spinous process of the T_2_vertebra, the examiner positioned themselves behind the subject and instructed them to bend their head. This positioning revealed two prominences at the base of the cervical region, representing the spinous process of the C_6_ and C_7_ vertebrae. The examiner then asked the subject to slowly tilt their head backward by applying slight pressure to these prominences. During this movement, one of the prominences (C_6_) no longer remained palpable, leaving only the single prominence of the C_7_ vertebra. By identifying the C_7_ spinous process, it became easier to locate the spinous processes of the T_1_ and T_2_ vertebrae by gently tracing down the spine. In the current study, after identifying the T_2_vertebra, the starting point of the kyphosis curve was marked using a landmark. To determine the T_12_ vertebra, the Hoppenfeld method, a widely utilized technique in various studies, was employed [24]. Individuals with a kyphotic angle greater than 46.83 degrees were classified as having an increased kyphotic deformity.

The rounded shoulder angle (RSA) was measured from the vertical posteriorly to a line connecting the C7 and acromial markers [25]. A shoulder angle of more than 52 degrees is considered RS deformity [26].

**Fig. 2:**
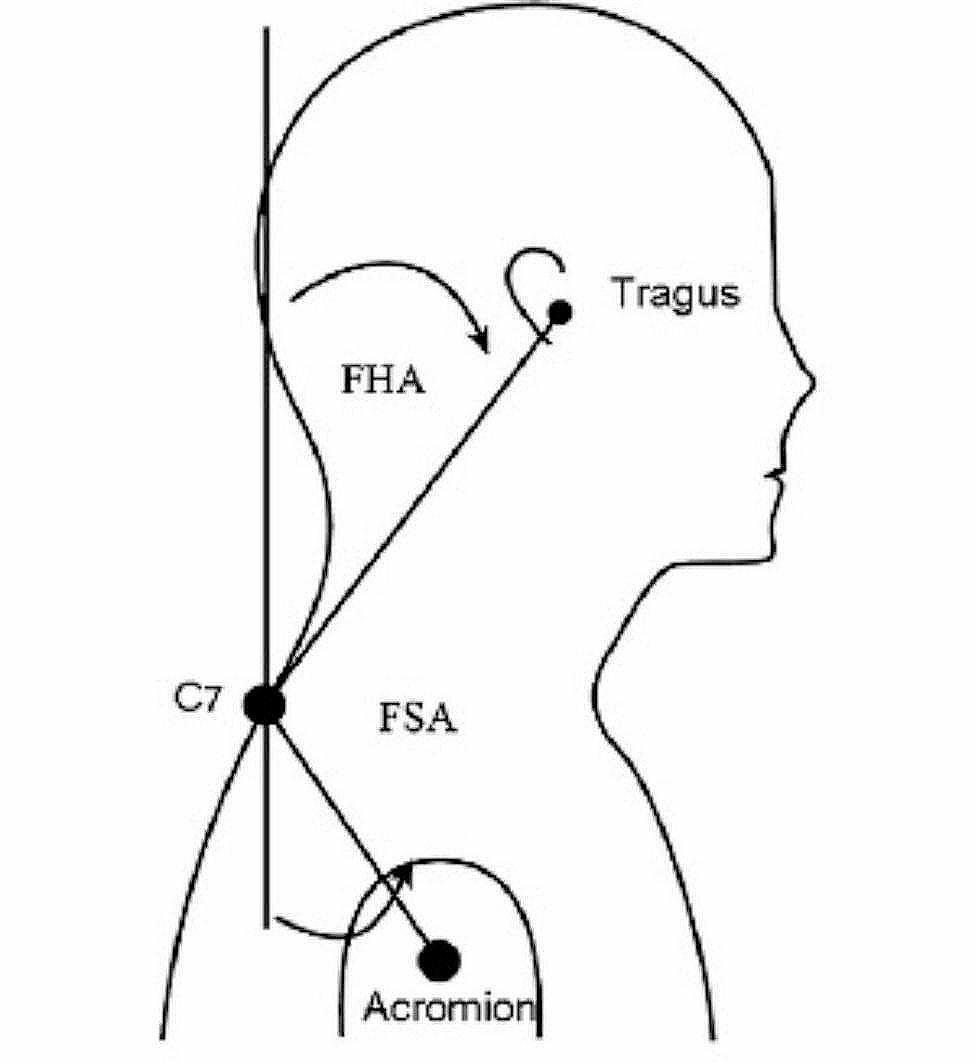
FHA measured from the vertical anteriorly to a line connecting the tragus and the C7 marker. The forward shoulder angle (RSA) was measured by measuring the angle between the vertical line running posterirly and the line connecting the markers at the C7 and acromial position [25]

Forward head angle (FHA) is measured from the vertical anterior to a line connecting the tragus and the C_7_ marker. The ideal angle of the head in this method is less than 36 degrees, and an angle of more than 46 degrees is considered an abnormality of the FH [26] (Fig. 2). Intraday reliability for FHA and FSA demonstrated acceptable within-day reliability (FHA = Intraclass Correlation Coefficient (ICC)_(2,1)_ = 0.92, Standard Error of the mean (SEM) = 2_ and RSA ICC_(2,1)_ = 0.89, SEM = 5_) based on this sub-sample [27].

### Interventions

#### Group A: corrective exercises

The CE exercises were done in the gym, and the corrective movements specialist was responsible for supervising their performance. Selected CE was designed to correct posture and address the mentioned abnormalities through stretching exercises for shortened muscles and strengthening exercises for individuals with weak muscles. These exercises included a 5-10-minute warm-up followed by stretching exercises for the chest, hip-flexor-psoas, upper trapezius, intercostal muscles, upper neck extensors, and then strengthening exercises for the shoulder protractors, deep neck flexors, lower neck extensors, and thoracic spine extensors.

#### Group B: Alexander’s technique

In the AT group, parents supervised the students’ exercises at home based on the explanations and training the corrective movements specialist provided in the first session. In the AT group, adolescents were taught the considerations and habits they should remember and focus on daily. These included teaching ergonomic considerations and individual postural habits during daily activities such as standing, walking, sitting, sleeping, reading, using a computer, and other repetitive and continuous activities performed during the day. Individuals were taught and reminded of these matters one to two times per week at school. Parents reported their implementation to the researcher. Parents played a fundamental role in this program and were responsible for reminding their children of correct postural habits and points through predetermined verbal instructions throughout the day. A poster containing images displaying proper postural habits while standing, sitting, and lying down was created and made available to individuals. This poster can be installed in a suitable location at home, allowing students to maintain correct posture by observing it regularly and remembering to do so.

#### Group C: Pilates exercises

PE was done in the gym. The Pilates instructor trained the subjects in the Pilates group, and a corrective movement specialist supervised the training during the exercises. In the first session of PE, the group received an explanation of the basic principles that were followed in all sessions. The following steps were taken in each PE session, including checking the posture (pelvis and spinal column), controlling breathing and standing in class (about 5 min), performing Pilates breathing and stretching exercises with coach explanations (about 10 min), performing modified specific exercises (about 40 min), and returning to the initial state (5 min). The exercises started at low levels and gradually progressed until the participants could control their spinal columns in various positions. The exercise intensity was personalized based on each participant’s pain and exercise tolerance threshold. As a result, with continued exercise, the participants could do more repetitions without feeling pain or fatigue. The exercises started with 8 repetitions and ended with 16 repetitions. Each session included new exercises in addition to those from the previous session.

### Data analysis procedure

We used the Shapiro-Wilk test to check for normality and Levene’s test to assess variance equality. The dependent t-test was used for within-group comparisons. We conducted one-way ANOVA with TukeyTest for multiple group comparisons to compare means pairwise. Data analysis was performed using SPSS software version 19 at a significance level of *p* < 0.05.

## Results

The statistical indices of age, height, and weight of the participants in different training groups are shown in Table 1.

**Table 1:** Statistical indices related to the participant’s age, height, and weight

Variable	group	Number	Mean ± SD
Age	CE	15	14.66 ± 0.72
	AT	15	14.06 ± 1.09
	PE	15	1.06 ± 15.00
Height	CE	15	7.79 ± 151.46
	AT	15	6.73 ± 150.66
	PE	15	8.25 ± 152.93
Weight	CE	15	7.69 ± 45.40
	AT	15	4.20 ± 42.53
	PE	15	6.96 ± 43.53

CE: Corrective Exercises; AT: Alexander’s Technique; PE: Pilates Exercises

The results of the dependent t-test showed that after 6 weeks of CE and AT, there was a significant decrease in FHA, RS, and kyphosis from the pre-test to the post-test phase (Table 2). However, in the PE group, a significant decrease in FHA, RS, and kyphosis from the pre-test to the post-test phase was not observed (Table 2).

**Table 2:** Comparison of FHA, RS, and kyphosis angle in participants before and after training using dependent t-test

	group	SD ± Mean		DF	T	Sig
		Pre-test	post-test			
FH	CE	50.86 ± 2.79	44.53 ± 3.50	14	5.02	*0.0001
	AT	52.93 ± 3.23	47.06 ± 3.51	14	6.14	*0.0001
	PE	51.86 ± 2.87	50.00 ± 4.10	14	1.70	0.110
RS	CE	61.60 ± 3.85	56.13 ± 3.29	14	4.36	*0.001
	AT	60.60 ± 3.79	55.86 ± 3.60	14	3.89	*0.002
	PE	62.46 ± 3.83	61.86 ± 3.04	14	0.54	0.598
Kyphosis	CE	52.06 ± 3.57	45.66 ± 3.47	14	4.81	*0.0001
	AT	51.06 ± 4.18	45.73 ± 2.73	14	4.06	*0.001
	PE	51.40 ± 3.88	50.40 ± 3.68	14	0.92	0.371

FH = Forward Head; RS: Rounded Shoulder; CE: Corrective Exercises; AT: Alexander’s Technique; PE: Pilates Exercises, DF = Degrees of freedom

The results of the one-way analysis of variance showed that after six weeks of PE, CE, and AT, a significant difference was observed in the FHA, RS, and kyphosis between the pre-test and post-test measurements (Table 3).

**Table 3:** Results of one-way analysis of variance (pre-test and post-test differences) for changes in the FHA, RS, and kyphosis in different exercise groups

	Variable	SS	DF	MS	F	Sig
FH	between groups	180.84	2	90.42	4.88	0.012
	Within group	776.80	42	18.49		
	Total	957.64	44			
RS	between groups	206.53	2	103.26	4.81	0.013
	Within group	900.26	42	21.43		
	Total	1106.80	44			
Kyphosis	between groups	245.37	2	122.68	5.26	0.009
	Within group	978.93	42	23.30		
	Total	1224.31	44			

FH = Forward Head; RS: Rounded Shoulder; DF: degrees of freedom; SS: Sum of Squares; MS: Mean Square

The Tukey post hoc tests indicate that no significant difference was observed between the CE group and the AT group in the FHA, RS, and kyphosis (Table 4). However, a significant difference was found between the CE group and the Pilates exercises group in the FHA, RS and kyphosis. Additionally, a significant difference was observed between the AT group and the Pilates exercises group in the FHA, RS, and kyphosis(Table 4).

**Table 4:** Tukey’s test findings to examine the differences in FHA, RS, and kyphosis

	Group I	Group J	MD	Std. E	Sig
FH	CE	AT	-0.46	1.57	0.953
		PE	-4.46	1.57	*0.018
	AT	PE	-4.00	1.57	*0.038
RS	CE	AT	-0.73	1.69	0.902
		PE	-4.86	1.69	*0.017
	AT	PE	-4.13	1.69	*0.048
Kyphosis	CE	AT	-1.06	1.76	0.818
		PE	-5.40	1.76	*0.010
	AT	PE	-4.33	1.76	*0.047

FH = forward head; RS: rounded shoulder; At: Alexander technique; CE: corrective exercises; PE: Pilates Exercises; MD: mean Differences(I-J); Std.E: Std.Error

## Discussion

To the authors’ knowledge, this is the first study that investigated comparing the effects of Pilates, corrective exercises, and Alexander’s technique on upper cross syndrome among adolescent girls’ students (ages 13–16): a six-week study. The main findings of this study were [1] Six weeks of CE and AT significantly reduced FHA, RS, and kyphosis in adolescent girls with UCS; [2] PE showed a reduction in FHA, RS, and kyphosis, but this reduction was not statistically significant; [3] There was a significant difference between the FH, RS, and kyphosis of girls in the PE, CE, and AT groups, with the CE and AT groups showing greater improvements than the PE group; [28] There was no significant difference between the mean FH of girls in the CE and AT groups; [29] There was a significant difference between the PE group and both the CE and AT groups in all three variables of FH, RS, and kyphosis. These findings suggest that CE and AT may be more effective than PE in improving UCS in adolescent girls.

The results of the dependent t-test showed that six weeks of AT significantly reduced FH angle, RS, and kyphosis in adolescent girls. Babaei et al. (2023) conducted a study on the effectiveness of the AT on the static and dynamic balance of young men with UCS. They found a significant improvement in both static and dynamic balance after the intervention [17]. Several studies have explored methods for correcting spinal abnormalities and improving posture in children and students. Cardon et al. (2001) proposed that teaching correct posture and continuous monitoring throughout the day in elementary school children is an appropriate approach [30]. Robbins (2009) observed a 75% reduction in musculoskeletal disorders by teaching students proper sitting [31]. Feingold (2002) found that teaching school children how to use backpacks correctly improved their posture, particularly in the shoulder position [32]. Heyman (2009) taught students various techniques, including correct posture, backpack use, and anatomical principles, resulting in improved posture and prevention of musculoskeletal disorders [33]. Hrysomallis et al. (2001) emphasized the importance of exercise frequency in correcting posture and recommended incorporating exercises into daily life [34]. The study focused on home teaching and retraining, aligning with previous research on the effectiveness of training programs in correcting UCS. These programs share similar effectiveness mechanisms but require time for the consolidation of good physical habits. Proper positioning, ongoing CE, and integrating exercise programs are necessary to correct physical issues. The findings emphasize the significance of teaching correct posture, providing education on backpack use, and integrating exercise programs to improve posture and prevent musculoskeletal disorders.

The results of the dependent t-test showed that 6 weeks of CE significantly reduced FH angle, RS, and kyphosis in adolescent girls. Abdullahzadeh et al.‘s (2019) study showed that eight weeks of CE based on NASM principles improved UCS in the experimental group compared to the control group [12]. Regular CE [13] and comprehensive CE along with teaching proper posture [14] had a significant effect on UCS [13, 14]. to obtain better results in their training programs, we suggest that experts pay attention to correcting abnormalities simultaneously as a new approach [35]. Zandi (2010) compared the effects of CE, postural retraining, and a combination of both on correcting UCS in adolescent girls. Significant differences were found in all variables between the CE group and the combined group. The postural retraining group showed improvements in kyphosis and forward head posture (FH), but no significant differences were observed in rounded shoulders (RS). These findings align with the present study, suggesting that the lack of stimulation of shoulder restricting muscles and the absence of attention to releasing myofascial tissues in the anterior chest region during postural retraining exercises may explain the absence of significant effects on RS [36]. The results of the studies showed that CE can improve FH posture [37], hyperkyphosis in girls [38] and boys [39], and spinal column posture [40]. These researchers recommend CE as a reliable method for improving spinal abnormalities, which can reduce the occurrence of future problems and discomfort.

Several studies have investigated the effects of PE on individuals with UCS, and the findings suggest that Pilates can provide significant benefits [41, 42]. In the present research, PE showed a reduction in FH angle, RS, and kyphosis from the pre-test to the post-test, but this reduction was insignificant. Recent studies have examined the effects of PE on individuals with various postural imbalances. Rahimi et al. (2022) surveyed middle-aged women with lower cross syndrome. They found that six weeks of PE enhanced the strength and efficiency of core stabilizer muscles, leading to improved function in both the upper and lower extremities [42]. Furthermore, de Araújo et al. (2012) found that the Pilates method was effective in reducing the degree of non-structural scoliosis, improving flexibility, and decreasing pain [41]. These findings highlight the potential benefits of PE in addressing various postural imbalances and enhancing function, alignment, and muscle strength. The results of these studies are consistent with the results of the present research. The studies support the effectiveness of Pilates exercises in targeting the muscles involved in UCS. These exercises help improve posture and muscular balance and promote body awareness. Future studies should include larger sample sizes, longer intervention durations, and control groups to establish the efficacy of PE in addressing UCS.

Previous studies support the effectiveness of EC and AT compared to PE in treating UCS. Combined exercises are more effective than independent CE and retraining [36, 43]. Some studies also report the effectiveness of physiotherapy and chiropractic interventions [18]. However, in some cases, no significant changes were observed, possibly due to qualitative evaluation methods, lack of posture assessment, or short exercise periods [44]. This study’s limitation is the small sample size of 45 students; only 15 students participated in each group. The study was conducted over six weeks, and if it had been conducted over a longer period, the effects of the interventions may have been more pronounced. Also, this study did not include a control group.

## Conclusion

The study highlights the potential benefits of Pilates, CE, and AT in addressing UCS in adolescent girls. The effect of Alexander’s technique and corrective exercises on forward head angle, rounded shoulder, and kyphosis abnormalities was almost similar and more effective than pilates exercises. These interventions offer promising ways to improve posture, alignment, muscular imbalances, and body awareness. Further research is needed to confirm these findings and explore the long-term effects on UCS in this population. Early interventions targeting UCS have the potential to foster healthy postural habits and enhance overall well-being among this group.

### Electronic supplementary material

Below is the link to the electronic supplementary material.

Supplementary Material 1

## Data Availability

Not applicable.
