# Supplementary material for: Comparing the effects of Pilates, corrective exercises, and Alexander’s technique on upper cross syndrome among adolescent girls student (ages 13–16): a six-week study
Source: BMC Sports Sci Med Rehabil. 2024 Jun 28;16:143. doi: 10.1186/s13102-024-00933-2 (PMC11214212; doi:10.1186/s13102-024-00933-2)
Supplement: Supplementary file 1 — Supplementary Material 1 [file 13102_2024_933_MOESM1_ESM.docx]

**Methods Reporting Checklist for Randomized Trial Based on CONSORT Guidelines**

**1. Title**

- "**A comparison of the effects of six weeks of Pilates exercises, corrective exercises, and Alexander's Technique on the upper cross syndrome in adolescent girls aged 13 to 16 years**"

**2. Introduction**

*- Clearly state the objective or research question of the study:*

The objective of this study was to examine a comparison of the effects of Pilates exercises(PE), corrective exercises(CE), and Alexander's Technique (AT) on upper cross syndrome(UCS) in adolescent girls aged 13-16 years: a six-week intervention study.

*- Provide background information on upper cross syndrome, its prevalence, and potential impact on adolescent girls:*

Upper crossed syndrome (UCS) is a common complication associated with modern technological life, characterized by weakened middle and lower trapezius, scalenes, deep cervical flexors, and serratus anterior muscles, as well as tightness in the upper trapezius, pectoralis minor and major, and levator scapulae muscles (3). The condition's name derives from the "X" appearance it creates through the top of the body. Muscle imbalances are the primary manifestation of UCS in both tonic and phasic muscles (4). Poor head and neck posture(5), abnormal breathing patterns, psychological issues in female adolescents, and inappropriate fitness methods are among the primary causative factors (6). Individuals with UCS often exhibit symptoms such as FH posture, RS, and restricted cervical spine mobility. These symptoms are closely linked to neck and shoulder pain, which can be persistent and difficult to manage (7). The long-term effects of chronic pain on mental health are significant, and UCS patients may experience negative emotions such as anxiety that can severely impact their quality of life (8). Abnormal postures associated with UCS can cause an increase in upper thoracic kyphosis, leading to a reduction in the thoracic cage's volume (9) and impeding the motion of muscles involved in active respiration (10). This can result in lower lung volumes and capacities, affecting thoracic expansion and alveolar ventilation. As a result, exercise tolerance and respiratory efficiency may decrease, and the partial pressure of oxygen may decline (11).

*- Explain why studying the effects of Pilates exercises, corrective exercises, and Alexander's Technique is important in addressing upper cross syndrome*:

In general, designing an effective method of treatment for this issue contributes significantly to saving a considerable portion of healthcare costs. Additionally, considering the prevalence of UCS among teenage girls due to improper use of mobile phones, tablets, and computers, not adhering to proper principles during walking, sitting on chairs, lying down, and carrying heavy backpacks, highlights the importance of conducting this research. So far, based on researchers' searches, no study has compared the effects of PE, CE, and AT on UCS in teenage girls. Therefore, the present study aims to address the question of whether six weeks of PE, CE, and AT can have different effects on UCS in 13 to 16-year-old teenage girls. Which exercises are more effective?

**3. Study Design**

*- Clearly state that the study is a randomized trial*

The study is a Quasi-experimental

*- Describe the design, such as parallel group design, randomization method, and allocation ratio*

This is a parallel group design. The present study was a Quasi-experimental type of research, and its statistical population consisted of 13 to 16-year-old female students in the city of Quchan. Among them, 45 students who were diagnosed with UCS were purposefully selected as samples and randomly assigned to three groups: PE (N=15), CE (N=15), and AT (N=15). The research design was a three-group design with pre-test and post-test in the exercise groups. The participants performed CE, AT, and PE for 60 minutes per session, three sessions per week, and for a duration of 6 weeks.

*- Indicate any blinding methods used, such as participant blinding, assessor blinding, or both*

In this study, we used blinding of the samples

4. Participants

*- Describe the target population, including the inclusion and exclusion criteria*

To be included in the study, participants had to have postural abnormalities such as kyphosis, FH, and RS simultaneously and express a willingness to participate. Observing any pathological symptoms, history of fractures, surgeries, joint diseases, and injuries in the cervical, thoracic, and lumbar spine region, skeletal-muscular imbalances, lower limb cross syndrome, having BMI outside the normal range, and having regular physical activity of at least 6 hours per week were considered as exclusion criteria from the study. Also, those who were aware of their condition before the study and had taken measures to correct the abnormalities based on medical advice were excluded from the study (due to the potential impact of previous interventions on the results of the current study).

*- Specify the total number of participants recruited and how they were selected*

The present study was a Quasi-experimental type of research, and its statistical population consisted of 13 to 16-year-old female students in the city of Quchan. Among them, 45 students who were diagnosed with UCS were Simple random sampling selected as samples and randomly assigned to three groups: PE (N=15), CE (N=15), and AT (N=15).

*- Provide demographic information such as age range, gender, and any other relevant characteristics*

The statistical indices of age, height, and weight of the participants in different training groups are shown in Table 1.

**Table 1.** Statistical indices related to the age, height, and weight of the participants.

| Variable | group | Number | Mean ± SD |
| --- | --- | --- | --- |
| Age | CE | 15 | 14.66±0.72 |
|  | AT | 15 | 14.06±1.09 |
|  | PE | 15 | 1.06±15.00 |
| height | CE | 15 | 7.79±151.46 |
|  | AT | 15 | 6.73±150.66 |
|  | PE | 15 | 8.25±152.93 |
| Weight | CE | 15 | 7.69±45.40 |
|  | AT | 15 | 4.20±42.53 |
|  | PE | 15 | 6.96±43.53 |

CE: Corrective Exercises; AT: Alexander's Technique; PE: Pilates Exercises

*- State any baseline characteristics used for randomization or adjustment*

Among them, 45 students who were diagnosed with UCS were purposefully selected as samples and randomly assigned to three groups: PE (N=15), CE (N=15), and AT (N=15).

5. Interventions

*- Describe each intervention group (Pilates exercises, corrective exercises, and Alexander's Technique)*

**corrective exercises**

Selected CE was designed to correct posture and address the mentioned abnormalities through stretching exercises for shortened muscles and strengthening exercises for weak muscles for the individual. These exercises included a 5-10-minute warm-up followed by stretching exercises for the chest, hip-flexor-psoas, upper trapezius, intercostal muscles, upper neck extensors, and then strengthening exercises for the shoulder protractors, deep neck flexors, lower neck extensors, and thoracic spine extensors.

**Alexander's Technique**

In the AT group, adolescents were taught the considerations and habits they should remember and focus on in their daily lives. These included teaching ergonomic considerations and individual postural habits during daily activities such as standing, walking, sitting, sleeping, reading, using a computer, and other repetitive and continuous activities performed during the day. These matters were taught and reminded to individuals in one to two sessions per week at school, and their implementation was reported to the researcher by parents. Parents played a fundamental role in this program and were responsible for reminding their children of correct postural habits and points through predetermined verbal instructions throughout the day. Additionally, correct postural habits of standing, sitting, and lying down were included in a poster with images and made available to individuals to be installed in a suitable location at home so that by observing it, students would always remember to maintain correct posture.

**Pilates exercises**

In the first session of PE, the basic principles of PE were explained to the group and an attempt was made to adhere to them in all sessions. The following steps were taken in each PE session, including checking the posture (pelvis and spinal column), controlling breathing and standing in class (about 5 minutes), performing Pilates breathing and stretching exercises with coach explanations (about 10 minutes), performing modified specific exercises (about 40 minutes), and returning to the initial state (5 minutes). The exercises started from low levels and gradually progressed until the participants were able to control their spinal column in various positions. The intensity of the exercises was determined for each participant based on their exercise tolerance threshold and pain. As a result, with continued exercise, the participants were able to do more repetitions without feeling pain or fatigue. The exercises started with 8 repetitions and ended with 16 repetitions. In each session, new exercises were added in addition to the previous session's exercises.

*- Clearly define the components of each intervention, including frequency, duration, and intensity*

60 minutes per session, three sessions per week, and for a duration of 6 weeks.

*- Specify any modifications or adaptations made to the interventions*

The interventions were done based on the group in which the samples should practice

*- Provide information on the training and qualification of the instructors delivering the interventions*

After several training sessions about the interventions that should be done and getting to know them completely, the trainers started to implement them

6. Randomization and Allocation

*- Explain the method of randomization used, such as computer-generated random numbers or randomization software*

computer-generated random numbers

*- Provide details on how participants were allocated to each intervention group, including any stratification factors or block randomization used*

45 students with UCS were randomly selected as a sample and were randomly divided into three groups: PE (N=15), CE (N=15).

*- State who performed the randomization and allocation procedures and whether they were blinded to participant information*

The researcher performed the randomization and allocation methods and was not blinded to the participants' information

7. Outcomes

*- Clearly state the primary and secondary outcomes measured in the study*

The primary outcomes of having UCS and the secondary outcome of UCS correction after the completion of the interventions

*- Describe how each outcome was assessed and measured, including any validated instruments or scales*

**Kyphosis measurement**

To measure the kyphosis angle, a flexible ruler was utilized, with the spinous process of T_2_ and T_12_ vertebrae serving as the starting and ending points of the kyphosis arc (34, 35). In order to locate the spinous process of the T_2_ vertebra, the examiner positioned themselves behind the subject and instructed them to bend their head. This positioning revealed two prominences at the base of the cervical region, representing the spinous process of the C_6_ and C_7_ vertebrae. By applying slight pressure to these prominences, the examiner then asked the subject to slowly tilt their head backward. During this movement, one of the prominences (C_6_) no longer remained palpable, leaving only the single prominence of the C_7_ vertebra. With the identification of the C_7_ spinous process, it became easier to locate the spinous processes of the T_1_ and T_2_ vertebrae by gently tracing down the spine. In the current study, after identifying the T_2_ vertebra, the starting point of the kyphosis curve was marked using a landmark. To determine the T_12_ vertebra, the Hoppenfeld method, a widely utilized technique in various studies, was employed (36). Individuals with a kyphosis angle higher than this amount (46.83 degrees) were considered to have increased kyphosis deformity.

**Measurement of the rounded shoulder and FH**

The rounded shoulder angle (RSA) was measured from the vertical posteriorly to a line connecting the C7 marker and the acromial marker (37). A shoulder angle of more than 52 degrees is considered RS deformity (38). Forward head angle (FHA) measured from the vertical anteriorly to a line connecting the tragus and the C7 marker. The ideal angle of the head in this method is less than 36 degrees, and an angle of more than 46 degrees is considered as abnormality of the FH (38) (**Figure 1**). Intraday reliability for FHA and FSA demonstrated acceptable within-day reliability (FHA = Intraclass Correlation Coefficient (ICC)_(2,1)_ = 0.92, Standard Error of the mean (SEM) = 2_ and RSA ICC_(2,1)_ = 0.89, SEM = 5_) based on this sub-sample(39).


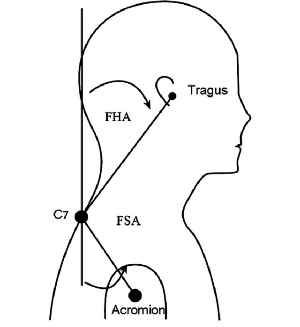


**Fig. 1.** FHA measured from the vertical anteriorly to a line connecting the tragus and the C7 marker. Forward shoulder angle (RSA) was measured from the vertical posteriorly to a line connecting the C7 marker and the acromial marker (37).

*- Provide details on the timing of outcome assessments (e.g., baseline, follow-up at six weeks)*

Six weeks after intervention

8. Sample Size Calculation

*- Explain how the sample size was calculated, including the power analysis and assumptions (e.g., effect size, alpha, beta)*

G-power

*- Justify the chosen sample size based on the expected treatment effects and statistical power*

The selected sample size is appropriate based on similar studies that have dealt with similar interventions. For each group, 15 people have been used in previous similar studies

9. Statistical Methods

*- Describe the statistical methods used to analyse the data, including any specific tests or models*

To examine the normality of the data distribution, the Shapiro-Wilk test was employed. To determine the equality of variances, Levene's test was used. For within-group comparisons (pre and post-test), the dependent t-test was utilized, while for between-group comparisons, the independent t-test was employed. To compare groups, a one-way analysis of variance (ANOVA) was conducted. The pairwise comparisons between the means of each group were conducted using Tukey's Test. Data analysis was performed using SPSS software version 19 at a significance level of p < 0.05.

*10. Ethics Approval and Informed Consent*

The Human Ethics Research Committee approved this study of the Sport Sciences Research Institute of Iran according to compliance with the Ethical Standards in Research of the Ministry of Science, Research and Technology, with the code IR.SSRI.REC. 1401.1724, as well as operating in accordance with the Declaration of Helsinki.

11. Data Collection and Monitoring

*- Describe how data were collected, including the data collection forms or electronic systems used*

The data is collected by the form

2. Trial Registration

*- Provide details of trial registration, such as the trial registration number and the registry where it was registered (e.g., ClinicalTrials.gov)*

the trial protocol for this study has been successfully registered in the Iranian Registry of Clinical Trials, with the assigned approval number IRCT20230810059106N1

**3. Results**

*- Present the main results of the study, including participant flow, baseline characteristics, and primary and secondary outcome data*

The results of the one-way analysis of variance showed that after six weeks of PE, CE, and AT, a significant difference was observed in the FHA (p=0.012), RS (p=0.013), and kyphosis (p=0.009) between the pre-test and post-test measurements.

The pairwise comparisons between the means of each group were conducted using Tukey's Test (Table 4). No significant difference was observed between the CE group and the AT group in the FHA (p=0.953), RS (p=0.902), and kyphosis (p=0.818).

However, a significant difference was found between the CE group and the Pilates exercises group in the FHA (p=0.018), RS (p=0.017), and kyphosis (p=0.010). Additionally, a significant difference was observed between the AT group and the Pilates exercises group in the FHA (p=0.038), RS (p=0.048), and kyphosis (p=0.047).

*- Report effect estimates and measures of precision, such as confidence intervals or p-values*

p ≤0.05 was used.

However, a significant difference was found between the CE group and the Pilates exercises group in the FHA (p=0.018), RS (p=0.017), and kyphosis (p=0.010). Additionally, a significant difference was observed between the AT group and the Pilates exercises group in the FHA (p=0.038), RS (p=0.048), and kyphosis (p=0.047).

**4. Discussion**

*- Interpret the results in light of the study aims and previous research*

In general, the results of previous studies are consistent with the present study and indicate the effectiveness of EC and AT compared to PE in treating UCS. The study's result emphasizes the greater effectiveness of combined exercises compared to independent CE and independent retraining (47, 52). On the other hand, some studies have reported the effectiveness of interventions such as physiotherapy and chiropractic (30), and in some studies, significant changes were not observed in the implementation of exercise protocols, retraining, and combination, which is likely due to qualitative evaluation methods, lack of assessment of posture or short exercise periods (53).

*-* *Address any limitations of the study, such as sample size, missing data, or potential biases*

1. Sample Size: One potential limitation of this study is the sample size. If the study included a small number of participants, it may limit the generalizability of the findings to a broader population. A larger sample size would provide more statistical power and increase the reliability of the results.

2. Short Duration: The study examined the effects of six weeks of intervention on upper cross syndrome. While this time frame is suitable for assessing short-term effects, it may not capture the potential long-term benefits or sustainability of the interventions. Future studies could consider longer follow-up periods to explore the lasting effects of Pilates exercises, corrective exercises, and the Alexander Technique.

*- Discuss the clinical implications of the findings and their relevance to the target population*

1. Individualized Treatment: The findings of this study can inform healthcare professionals, such as physical therapists and exercise specialists, about the efficacy of Pilates exercises, corrective exercises, and the Alexander Technique in managing upper cross syndrome in adolescent girls. This knowledge can help guide the development of personalized treatment plans based on the needs and preferences of individual patients.

2. Early Intervention: The study focused on adolescent girls aged 13 to 16 years. These findings can contribute to the identification and early intervention of upper cross syndrome in this specific age group. By recognizing the potential effects of these interventions at an early stage, healthcare professionals can provide targeted interventions to mitigate or prevent the progression of upper cross syndrome.

3. Integration with Existing Treatment Approaches: The study examined the efficacy of three different intervention approaches. The findings can facilitate the integration of Pilates exercises, corrective exercises, and the Alexander Technique with existing treatment modalities for upper cross syndrome. This integrated approach can enhance the multidisciplinary management of this condition and potentially improve patient outcomes.

4. Patient Education: The study's results can be utilized to educate adolescent girls, their parents, and healthcare providers about the potential benefits of Pilates exercises, corrective exercises, and the Alexander Technique. By raising awareness and understanding about these interventions, patients and their caregivers can make informed decisions about their treatment options.

**15. Conclusion**

*- Summarize the main findings of the study, highlighting the effects of Pilates exercises, corrective exercises, and Alexander's Technique on upper cross syndrome in adolescent girls*

The results of the dependent t-test showed that 6 weeks of CE and AT significantly reduced forward head angle (FHA), rounded shoulder (RS), and kyphosis in adolescent girls. PE showed a reduction in FHA, RS, and kyphosis from the pre-test to the post-test, but this reduction was not significant. One-way ANOVA results showed a significant difference between the FH angle, RS, and kyphosis of girls in PE, CE, and AT groups. Toki test results showed no significant difference between the mean of FH angle in girls in the CE and AT groups. Toki test results showed there was a significant difference between the PE group and both CE and AT groups in all three variables of FHA, RS, and kyphosis.

*- Provide recommendations for future research or clinical practice based on the study results*

Further research is needed to confirm these findings and explore the long-term effects of these interventions on UCS in this specific population. Overall, the study contributes to the existing knowledge on non-invasive interventions for improving postural imbalances in adolescent girls and lays the foundation for potential applications in clinical practice and physical education settings.

**This Methods section reporting checklist is based on the CONSORT guidelines and should be used to ensure the comprehensive reporting of the randomized trial.**
